# Enzymatic purification of microplastics in soil

**DOI:** 10.1016/j.mex.2021.101254

**Published:** 2021-01-31

**Authors:** Oluchi Mbachu, Graham Jenkins, Chris Pratt, Prasad Kaparaju

**Affiliations:** a School of Engineering and Built Environment, Griffith University, Nathan Campus, QLD 4111, Australia; b School of Environment and Science/Australian Rivers Institute, Griffith University, Nathan Campus, QLD 4111, Australia

**Keywords:** Microplastics, Enzymatic digestion, Organic materials, Soil

## Abstract

Accurate and effective analysis of microplastics depends on the purification of any biogenic matter present in the environmental sample. Within the soil environment, the presence of biogenic materials (living and non-living) obstruct reliable identification of microplastics. However, while conventional acidic, alkaline, and wet peroxidation methods are often used for microplastics purification, they can result in significant alteration to the polymer integrity. To overcome this issue of polymer damage, we have developed a more efficient protocol using enzymes to eliminate organic materials from soil media without affecting the synthetic polymers. This method describes a simple enzymatic protocol that eliminates the organic matter based on the application of enzymes that target the three natural components of lignocellulosic biomass (cellulose, hemicellulose, and lignin). The enzymatic protocol introduced in this study allows for the use of an oxidizing agent for the pre-treatment of samples and density separation step. Additionally, this method was used to determine the enzymatic digestion efficiencies of soil organic matter and recovery rates of microplastics. Finally, the recovered microplastics were characterized by techniques including stereomicroscopy, FT-IR, and SEM.

This method proved to be effective in reducing approximately >90% of organic materials in soil samples. After showing a high digestion efficiency the method was applied to spiked microplastic soil samples where high recovery rates were established and plastic characteristics were conserved. Despite being a time consuming and expensive method when compared to other purification methods. The key benefits of this methodology are:

• Minimal sample preparation
• Representative purification of microplastic from diverse soil media; and
• Most importantly, preservation of polymer integrity aiding requirements needed for particle identification (e.g.,% mass polymer remaining, SEM images revealing no obvious polymer morphology change after enzyme treatment).

## Graphical abstract

**Figure:**
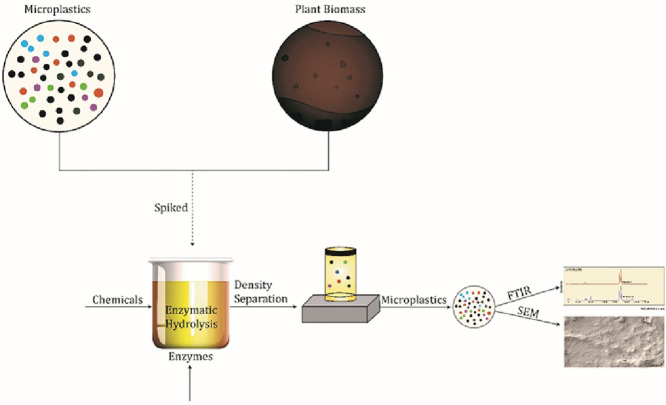

**Specification Table**

**Table:** 

Subject Area	Environmental Science
More specific subject area	*Soil microplastic contamination*
Method name	*Enzymatic purification of soil microplastic*
Name and reference of the original method	Löder, M.G., et al., *Enzymatic purification of microplastics in environmental samples.* Environmental science & technology, 2017. **51**(24): p. 14283–14292
Resource availability	*N/A*

## Background

Biogenic natural organic materials are a constraint in microplastic identification particularly due to a similar density range (1.0–1.4 g/cm^3^) to common environmental microplastics like PET (density around 1.3 g/cm^3^) and nylon (1.15 g/cm^3^) [1]. Typically, during microplastic extraction (density separation), organic materials float into the supernatant impeding microplastic identification and characterization. The use of Raman spectroscopy for the identification of organic-bound microplastics in untreated samples has therefore produced false signals in many instances. Hence, the elimination of biogenic organic material is a crucial requirement before microplastics analysis can be carried out for many matrices.

Enzymatic digestion methods are known to be effective and efficient for biogenic materials removal with little to no impact on microplastic particles. However, most reports on enzymatic digestion have focused on marine water and biological tissue samples with Löder et al. [2] developing a universal enzymatic protocol for marine planktonic samples. von Friesen et al. [3], and Löder et al. [2] conducted purification studies for the digestion of bivalve tissues and plankton samples with enzymes, respectively. They found that purification with enzymes achieved 97.7% and 98.3% digestion efficiencies. Cole et al. [4] also developed and tested enzymatic digestion protocols for seawater samples using Proteinase-K and reported a digestion efficiency of >97%. Based on these studies, the use of enzymes for removing soil organic materials may offer a promising approach for microplastic analysis of soil media.

One aspect that needs to be considered in applying enzymatic digestion methods to soils is the specificity of the enzymes used. In previous studies focusing on marine environmental samples, specific enzymes that target specific organic compounds have been used, optimizing enzymatic digestion efficiencies for the target compounds. For example, the enzymatic protocol presented by Löder et al. [2] uses protease, cellulase, and chitinase enzymes which is adequate for breakdown of marine planktonic samples. However, this enzyme recipe is not likely to be appropriate for soils which contain a much broader suite of organic compounds. Here, we test a novel enzymatic digestion approach for removing organic materials from soil media involving a sequential digestion procedure comprising oxidizing reagents in conjunction with enzymes targeting the most commonly encountered degradation-resistant organic materials in soils (i.e., cellulose, hemicellulose, proteins, and lipids). We also evaluate the potential impacts of the digestion method on the integrity of commonly- encountered microplastic polymers (i.e., low- density polyethylene, and polypropylene).

## Required reagents and equipment

• Sodium dodecyl sulphate 10%; Sigma-Aldrich (NSW, Australia)
• Dulbecco's phosphate-buffered saline solution; Sigma-Aldrich (NSW, Australia)
• Deionized water
• 30% w/w Hydrogen peroxide (H_2_O_2_) aqueous solution (AR); Chem-supply (SA, Australia)
• Anhydrous salt of zinc chloride (ZnCl_2_) (LR); Chem-supply (SA, Australia). 1.7 g/cm^3^ Saturated solution of ZnCl_2_ was prepared as described previously [5] and was maintained constantly in this study.
• Incubator; (Incucell LSISB2V|IC55)
• Drying ovens
• Muffle furnace; (JSR electric muffle furnace)
• Stainless steel soil sieves
• 11 µm nylon net filter (47 mm, Merck Millipore Ltd)
• Vacuum filtration flask
• Weighing balance;(Ohaus analytical balance SPX2202).
• 250 ml borosilicate glass stoppered incubation bottle

## Materials

• Lophostemon confertus plant biomass used as organic materials
• Soil; River Sands PTY LTD, (Brisbane Australia)
• Enzymes (Cellulase, Hemi-cellulase, Lipase, and Protease); Novozymes (NSW, Australia)
• Recycled (re-grind) plastic particles; Resitech Industries (Brisbane Australia)

## Analytical instrumentation

Scanning Electron Microscopy (SEM) and Fourier-transform infrared spectroscopy (FTIR) Imaging Methods were used for micro-surface and spectral imaging of microplastics, respectively. In this study, the SEM used for microplastic imaging consisted of using a Leica EM ACE600 coater, attached to 13 mm round SEM aluminum stubs using adhesive carbon tabs. Micrographs were collected at 3 kV using the secondary electron detector to show surface topography on a Zeiss Sigma Field Emission SEM.

Spectral images were collected on a Thermo Nicolet iS50 FTIR spectrometer equipped with a multi-bounce Ge ATR cell. A total of 256 spectra were collected with a resolution of 4cm^−1^. The resultant data was corrected in Thermo Omnic with an ATR correction to account for the variable degree of penetration of the evanescent wave into the sample. No smoothing was applied to the spectra.

## Digestion protocol

### Plant sample preparation

(1) Rinse plant leaves including its petiole with denoised water
(2) Transfer rinsed plant materials to a convection oven and dry for 7days at 40 °C
(3) Mill dried plant biomass using a coffee grinder and sieve to obtain particle size < 2 mm
(4) The ground plant material was stored in preparation for spiking the soil matrix tested in this method development work.

### Soil preparation

Biogenic-free soil was selected for use in this method development work, to control and measure biological inputs and evaluate the effectiveness of the technique.

(1) Homogenize obtained soil samples and sieve to less than 2 mm.
(2) Air dry at a temperature of 105 °C and calculate moisture content.
(3) Heat soil samples to a temperature of 550 °C to oxidize any biogenic impurities including organic matter.
(4) Store resulting clean soil sample at room temperature in a glass jar covered with aluminum foil to prevent plastic contamination.

### Plant material pretreatment

To increase the potential for comparability and to evaluate the effectiveness of enzymes on the dissolution of soil organic materials, plant materials were separated into two samples; pretreated and untreated.

(1) Add 50 g of milled plant material into a beaker placed on an analytical balance and weigh
(2) Add 100 ml of 30% v/v H_2_O_2_ and incubate at 60 °C for 24 h
(3) Vacuum filter to obtain digestate, then allow to air dry for 48 h
(4) Perform compositional analysis on the solid fraction of both untreated and pretreated plant materials

### Contamination mitigation

To reduce the risk of sample contamination, several actions were taken.

(1) During soil sample preparation only cotton laboratory coats were worn.
(2) Clean soil samples were covered with aluminum soil to prevent airborne microplastic contamination.
(3) Before use, all plastic particles and laboratory wares were washed and rinsed with deionized water. Plasticware was not used in this study.
(4) All reagents and chemicals were filtered before use through a 0.45 µm Glass fibre filter paper (47 mm) (Merck Millipore, Australia).
(5) Workbenches were wiped thoroughly with an isopropyl alcohol solution to any remove impurities.

## Enzymatic protocol

The following detailed steps were carried out for the sequential enzymatic purification of organic material as follows:

### Step 1: Sodium dodecyl sulphate treatment

(1) Transfer 1-g biomass (dry weight) of samples into a 250-ml glass stoppered bottle and weigh, add 50 ml of 10% sodium dodecyl sulphate solution.
(2) Agitate the mixture to achieve homogeneity.
(3) Place mixture in an incubator for 24 h at 50 °C.
(4) Allow cooling of glass bottle and contents for 15 mins and record weight.
(5) Decant liquid using vacuum filtration and transfer any residue on the filter paper back into the bottle for the following treatment step.

### Step 2: Cellulase and hemicellulase treatment

(1) Load Cellulase (Cellic) and Hemicellulase (Viscoferm) in the ratio of 5:1 ml into the glass stoppered bottle with wet biomass.
(2) Add 20 ml of acidic phosphate-buffered solution (PBS) adjusted to a pH 5 by adding hydrochloric acid and weigh the mixture.
(3) Place mixture in the incubator for 4 days at 50 °C. After the specified reaction time, allow the glass bottle to cool for 15 mins, and record the weight.
(4) Denature enzyme activity by placing in a water bath of temperature 50 °C for 15 min.
(5) Decant the liquid (enzyme and buffer solution) and transfer the solid fraction on filter paper into the stoppered bottle for the next enzyme treatment.

### Step 3: lipase treatment

(1) Add 5 ml of lipase (Lecitase) to the residue from cellulase and hemicellulase treatments.
(2) Add 20 ml of acidic PBS solution adjusted pH of 5 with HCl and weigh.
(3) Stir and place the mixture into the incubator at 50 °C for 3 days. After the specified reaction time, allow the glass bottle to cool for 15 mins, and record the weight.
(4) Place in a water bath at 50 °C to denature enzyme activity for 15 min.
(5) Decant the liquid (enzyme and buffer solution) and transfer the solid fraction on filter paper into the stoppered bottle for the next enzyme treatment.

### Step 4: protease treatment

(1) Add 5 ml of protease (Alcalase), into the glass stoppered bottles and 20 ml of alkaline PBS solution adjusted pH of 9 with NaOH and weigh.
(2) Incubate the mixture for 5 days at a reaction temperature of 50 °C.
(3) Cool glass bottle for 15 mins and weigh contents.
(4) Place in a water bath at 50 °C to denature enzyme activity for 15 min.
(5) Decant off the liquid and transfer solid fraction on filter paper into the glass bottles for further chemical treatments.

### Step 5: hydrogen peroxide treatment

(1) Add 30 ml of 30% w/w H_2_O_2_ to the residue, weigh and place in an oven at 60 °C for 24 h.
(2) After oxidation, allow to cool and weigh glass contents
(3) Vacuum filter off liquid samples and air-dry digestate on filter paper for 48 h.

**Note**: The residue left after the digestion procedure was weighed to calculate enzymatic digestion efficiency.

1 Calculate the enzymatic digestion efficiency using a modified digestion efficiency formula [6].
  $$\text{Digestion}\;\text{efficiency}\;\left(\%\right)\;=\;\frac{Wi-Wf}{Wi}\times 100$$

Where: W*_i_* = Initial weight of organic materials, W*_f_* = Final weight of organic materials (digestate), and W*_f_* = Weight of dry filter paper with retentate – weight of dry filter paper. Weights of dry filter paper (procedural blanks) were recorded before filtration.

**Fig 1:**
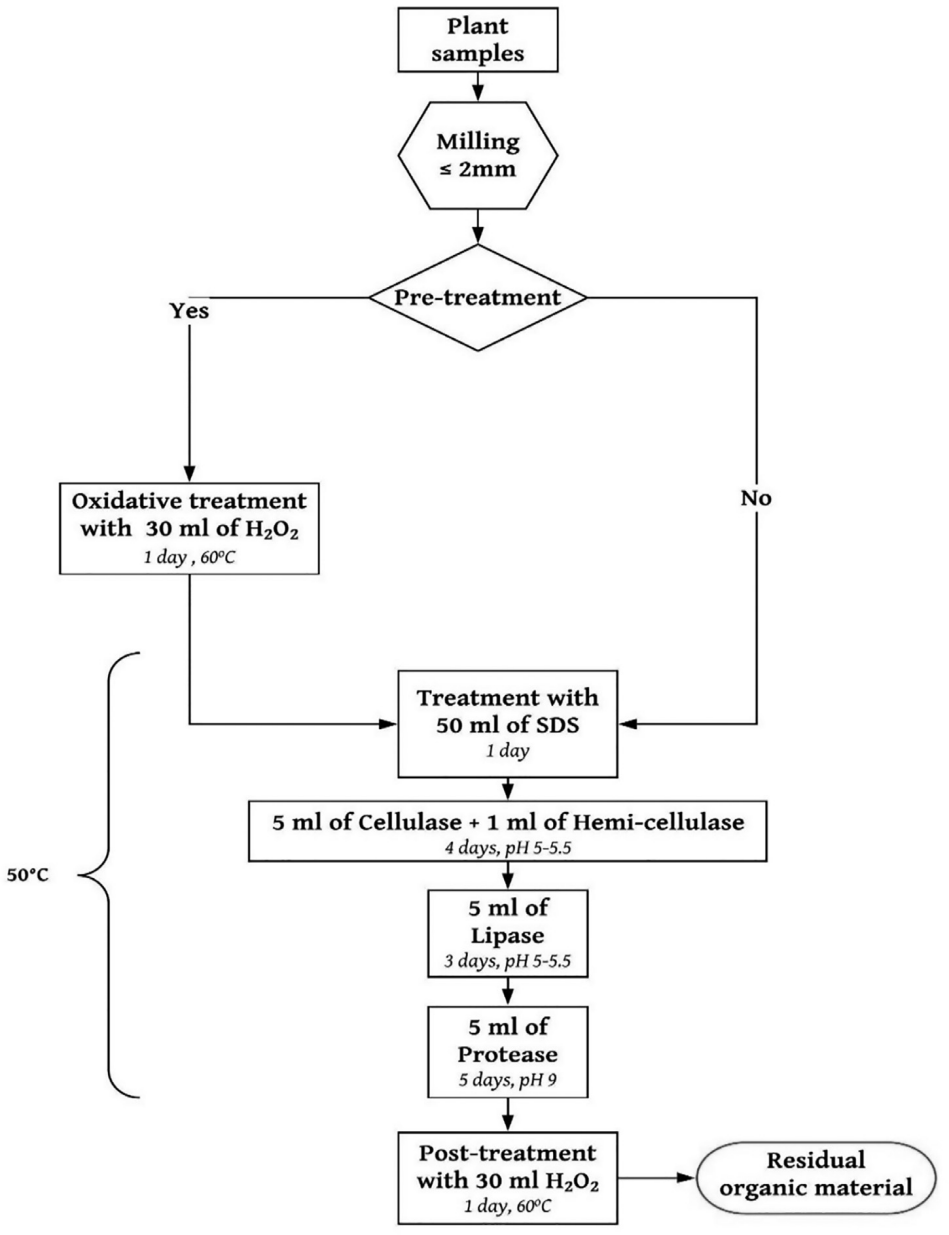
Schematic presentation of the sequential enzymatic purification procedure for organic materials.

**Note**: Enzymatic protocol was performed using three parallel sample treatments: pre-treated, untreated, and blank. Each treatment was performed as singlicates for each plant material (*n* = 6). The description of each treatment is given in Table 1. The purification assessment commenced according to the enzymatic protocol as shown in Fig. 1. After the digestion procedure, the organic matter residue was air-dried, and weights were determined gravimetrically with an Ohaus analytical balance (model SPX2202). Our results show that the enzymatic digestion rates from pre-treated, untreated, and blanks samples were 94%, 73%, and 0% respectively (see SI Table S1). For the effective enzymatic removal of biogenic matter from the soil, pre-treating samples with H_2_O_2_ was more effective than untreated samples. The oxidizing agent (H_2_O_2_) was able to break down recalcitrant compounds making them accessible for the enzymatic breakdown. It is imperative to pre-treat samples at 60 °C, as higher temperature affects polymer structure.

**Table 1:** Description of sample treatments.

	Control	Untreated	Pre-treated
Plant biomass (1.00 g)	*✓*	*✓*	*✓*
Pre-treatment (H_2_O_2_)	x	x	*✓*
Enzymes (Cellulase, hemicellulase, lipase, protease)	x	*✓*	*✓*
Chemicals (phosphate-buffer, SDS, H_2_O_2_)	x	*✓*	*✓*

## Effect of enzyme digestion on polymer integrity

For the polymer integrity analysis, microplastic particle measurements were performed using LDPE pellets, LDPE fragments, and PP fragments with an average particle size of 4.5 mm. The analysis was aimed at understanding the effect of the developed enzyme digestion procedure on microplastic physical and chemical properties.

**Fig 2:**
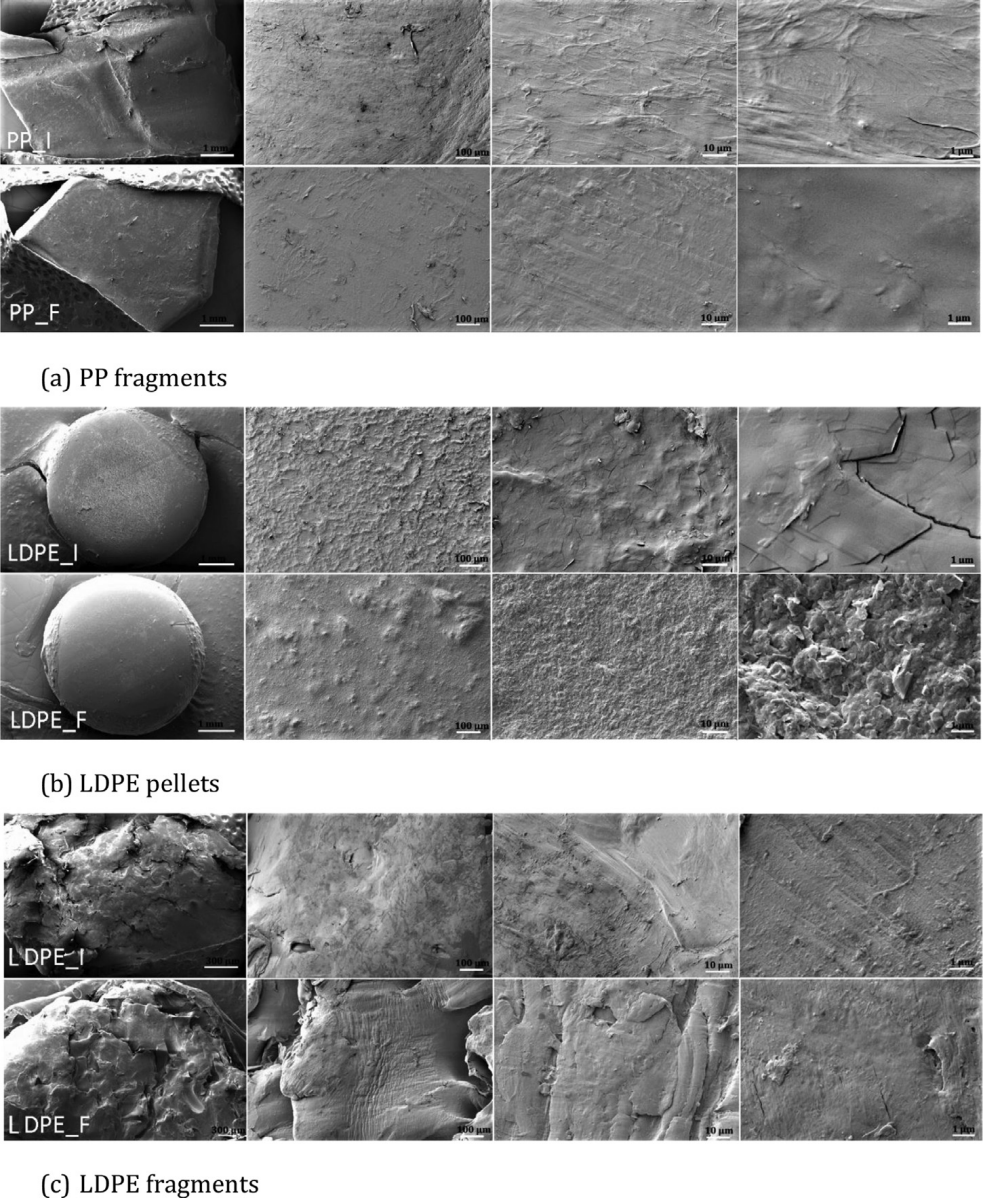
SEM images of microplastics before and after the enzymatic protocol.
(7) Finally, to determine the change in spectra of the chemical composition of the three polymers before and after the enzymatic protocol, FTIR coupled with ATR was conducted using sample #9. In the FT-IR spectrum of LDPE pellets, LDPE fragments, and PP fragments (Fig 3), an absorption peak appeared in the region of 4656–4511 cm^−1^ in both treated and untreated samples, corresponding to the OH-(hydroxyl) groups. The similar absorption peaks in control and treated samples of each MP particle indicates a strong overlap, suggesting that enzyme hydrolysis had no significant effect on the chemical composition of MPs. The presence of the OH-(hydroxyl) groups which represent hydrolytic gain can be attributed to the microplastic preparation steps of soaking and washing. Initial pictorial Images of the particles used are provided in SI Figure S1–3.

(1) Microplastic samples were placed into 5 ml glass vials using 10 vials for each polymer sample and then subjected to the enzyme protocol.
(2) The first microplastic samples from each polymer group, identified as sample #1 were collected after the first treatment step (H_2_O_2_), sample #2 were collected after SDS treatment. Similarly, samples #3, #4, #5, and #6 were collected after cellulose, lipase, protease, and H_2_O_2_ treatments respectively.
(3) Samples #7, #8 and #9 were collected at the end of the enzymatic protocol. These samples were representative of the complete enzymatic procedure and gave insight into potential sample degradation during the digestion protocol.
(4) After each sample collection, the microplastic mass was weighed with an Ohaus analytical balance (model PA214C) with readability of 0.01 mg.
(5) Size distribution and colour change of particles were performed using a prism optical stereomicroscope with a digital camera. According to the results, the size distribution and colour of observed particles remained intact.
(6) Microscopy analysis: SEM microscopy was used for surface image analysis of particles at the micro-levels following subjection to the digestion method (Fig 2). The SEM showed no detectable difference between treated and untreated samples. The SEM of the treated samples (i.e. samples that underwent enzymatic digestion) shows minor surface smoothness (Fig 2A). From obtained images, it is clear that the enzymatic protocol does not alter the microplastic surface morphology.

**Fig 3:**
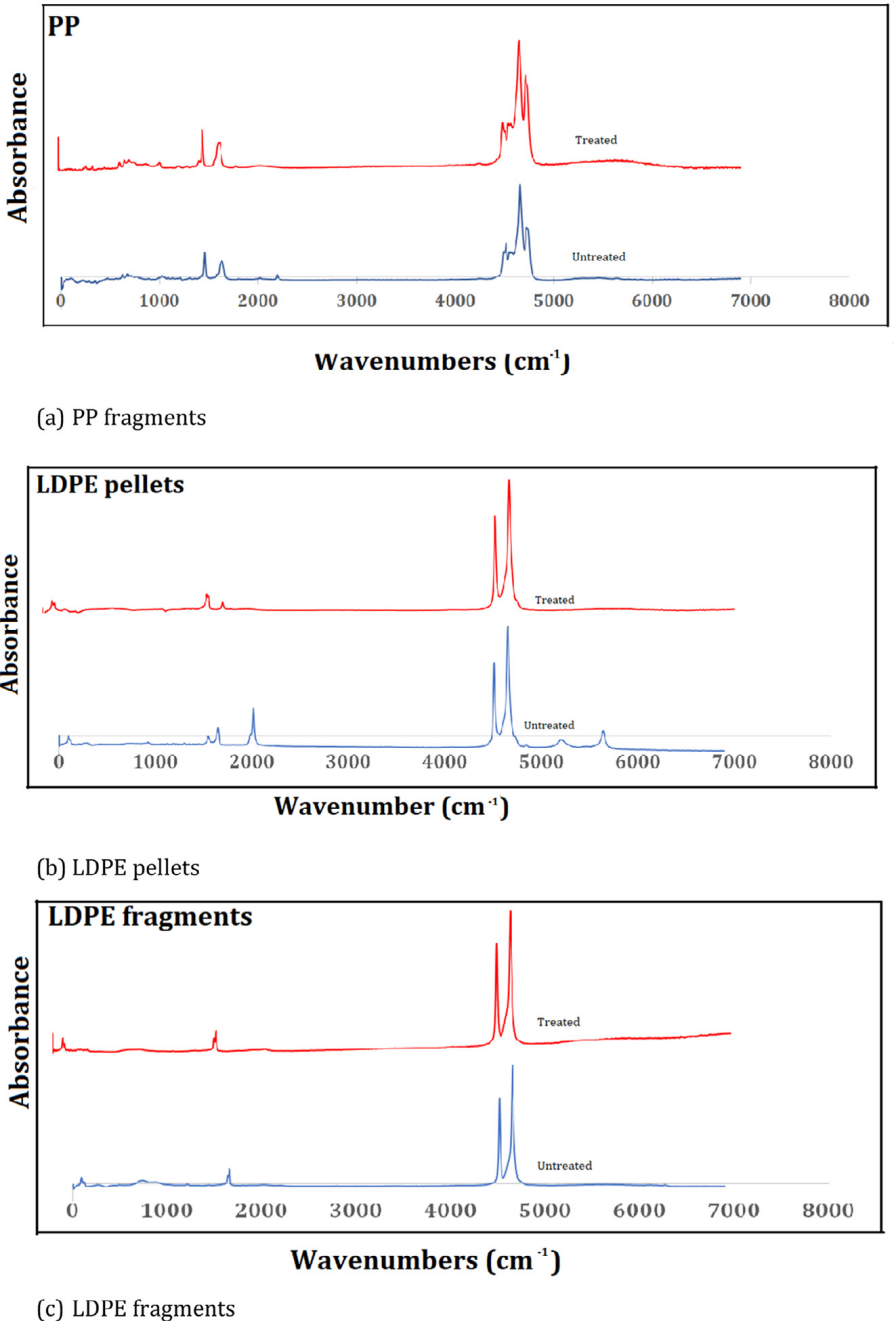
FT-IR spectrum of the control and treated microplastic particles.

## Method validation

**Fig 4:**
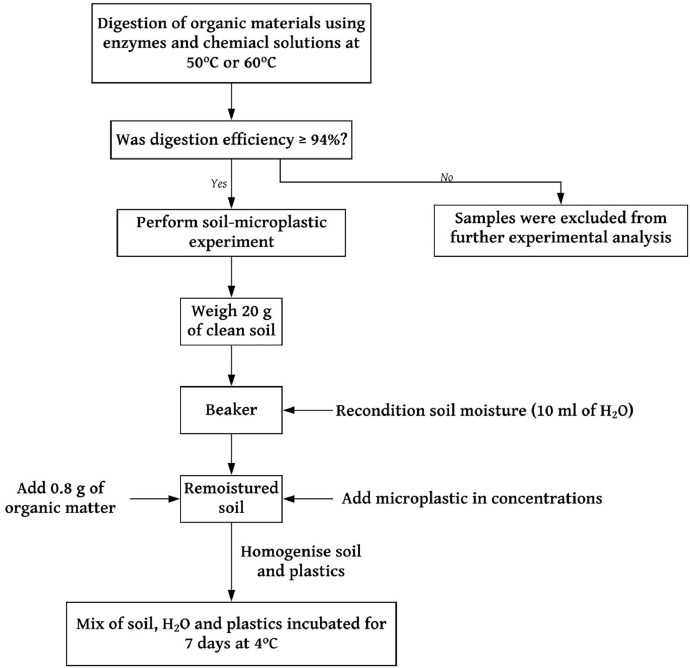
Stepwise approach for soil microplastic establishment employed in this study

**Note**: Three replicates of each polymer were prepared in addition to the three control samples (i.e., no microplastics).

To evaluate that the presence of soil and microplastic particles would not inhibit the organic material removal process and the recovery potential of microplastic, soil samples were spiked. Digestion and recovery experiments were performed as established by the stepwise decision tree presented in Fig. 4 and LDPE, and PP polymers with 0.045–2 mm size range obtained via dry sieving. Details of each polymer are given in SI Table S2.

(1) Add 0.8 g of organic matter and 2.5% (0.5 g) of each polymer to 20 g of clean soil (SI Table S3).
(2) Recondition soil mixture to 50% initial moisture content, stir, and then incubated for 7 days at 4 °C to obtain a dry and homogenous sample.
(3) Perform enzymatic digestion protocol as described above and calculate digestion efficiencies gravimetrically.

Experiments to evaluate the extraction potential of spiked microplastics began at the end of the enzymatic digestion.

(1) Add 100 ml of ZnCl_2_ solution to each beaker containing microplastics, sand, and residual organic matter.
(2) Agitate mixture on an electric shaker at 800 rpm for 15 min
(3) Let sit overnight, and then decant supernatant and vacuum filter through an 11 µm nylon membrane filter paper.
(4) Repeat step at least thrice until no microplastics is visible to the eyes in the supernatant.
(5) Allow the residues on the filter paper to air-dry at room temperature for 48 h, and record weights.
(6) Recovery rates were calculated as described by Zhang et al. [7] using the formula:
  $$\text{Recovery}\;\text{rates}\;\left(\%\right)\;=\;\frac{Wp}{Wadded\;}\;\times 100$$

W_t_ = weight of total flotation (W_1_ –W_2_), W_p_ = weight of microplastic floatation (W_g_ –W_control_), and W_control_ = weight of floatation collected from blank samples with no addition of microplastics. Following microplastic addition, enzymatic digestion rates decreased when compared to blank samples (SI Table S3). Our results indicate a relationship between particle morphology and enzymatic digestion efficiencies. The spherical particles for example LDPE pellets resulted in a mean enzymatic digestion efficiency rate of 93.1 ± 4.7% while the fragmented particles of LDPE and PP resulted in mean enzymatic digestion efficiencies of 72.6 ± 4.4% and 86.4 ± 5.9% respectively. Additionally, the determined recovery rates for the LDPE pellets, LDPE fragments, and PP fragments resulted in a mean value of 91 ± 0.1%, 147 ± 0.1%, and 108 ± 0.2% respectively (see Table 2). Notably, the recovery rates of microplastics correspond with the enzymatic digestion efficiency of soil organic matter. For example, LDPE fragments had the highest recovery rate yet the lowest digestion efficiency, indicating the presence of undigested materials in recovered particles.

**Table 2:** Summary of the soil-microplastic validation experiment.

Treatments	Recovery rate			Digestion efficiency (%)
	Wt of total floatation (Wt) (g)	Microplastics floatation (Wp) (g)	Mp Recovery (%)	
LDPE_p_	0.62 ± 0.1	0.46 ± 0.1	91 ± 0.1	**93.1 ± 4.7**
LDPE_f_	0.90 ± 0.1	0.74 ± 0.1	147 ± 0.1	72.6 ± 4.4
PP	0.70 ± 0.1	0.54 ± 0.1	108 ± 0.2	86.4 ± 5.9
Blank	–	–	–	**94.6 ± 0.7**

Wc; Wt of control floatation (0.16) was constant. LDPE_p_= pellets_,_ LDPE_f_ =fragments, Values in bold represent optimum digestion efficiency.

## Conclusions

In this study, for the first time, a method of microplastics purification from soil media by using enzymatic hydrolysis method was developed and validated. The proposed method is simple and polymer conserving. The validation of the enzymatic protocol demonstrates basic analytical performance parameters such as reproducibility, trueness (recovery), precision, and accuracy that can be achieved when the enzymatic digestion protocol presented above is followed.

## Appendix Supplementary materials

Image, application 1
