# Supplementary material for: Enzymatic purification of microplastics in soil
Source: MethodsX. 2021 Jan 31;8:101254. doi: 10.1016/j.mex.2021.101254 (PMC8374289; doi:10.1016/j.mex.2021.101254)
Supplement: Supplementary file 1 [file mmc1.docx]

**Supplementary information**

| Samples | initial Wt of glass bottle + OM  (dry weight) | Wt of H2O2 pre-treatment | Wt after pre-treatment | Wt of SDS | Wt after SDS | Wt of cellulose and hemicellulose | Wt after cellulose and hemicellulose | Wt of lipase | Wt after lipase | Wt protease | Wt after protease | Wt H2O2 | Wt after H2O2 | Final Wt of glass bottle + OM  (dry weight) | Final mass of OM | Digestion efficiency (%) |
| --- | --- | --- | --- | --- | --- | --- | --- | --- | --- | --- | --- | --- | --- | --- | --- | --- |
| LC Blank | 256.71 |  |  | 256.71 | 256.71 | 256.71 | 256.71 | 256.71 | 256.71 | 256.71 | 256.71 | 256.71 | 256.71 | 256.71 | 1.00 | 0 |
| LC Pre | 246.82 | 291.46 | 291.2 | 269.26 | 269.19 | 284.51 | 284.35 | 282.76 | 282.53 | 278.5 | 278.16 | 277.5 | 276.93 | 245.86 | 0.06 | 94 |
| LC NT | 257.82 |  |  | 277.36 | 277.29 | 296.12 | 295.95 | 293.73 | 293.46 | 291.54 | 291.04 | 291.97 | 291.25 | 257.11 | 0.27 | 73 |

Table S1: Enzymatic results of plant material digestion; all weights are in grams (g)

*LC*: Lophostemon confertus; NT: not treated; Pre: Pre-treated


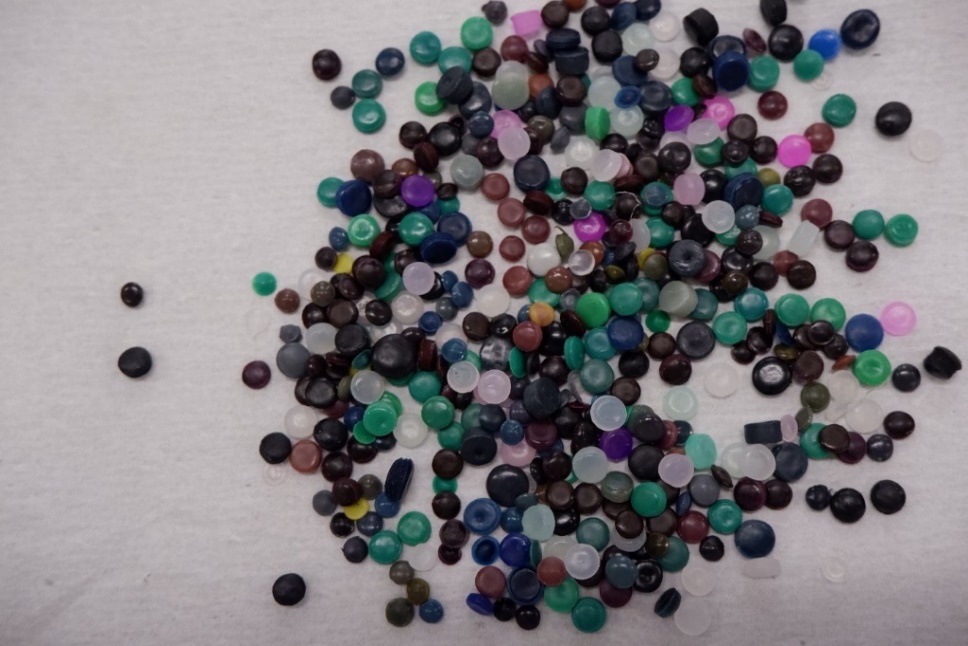


Figure S1: LDPE pellets used in this study


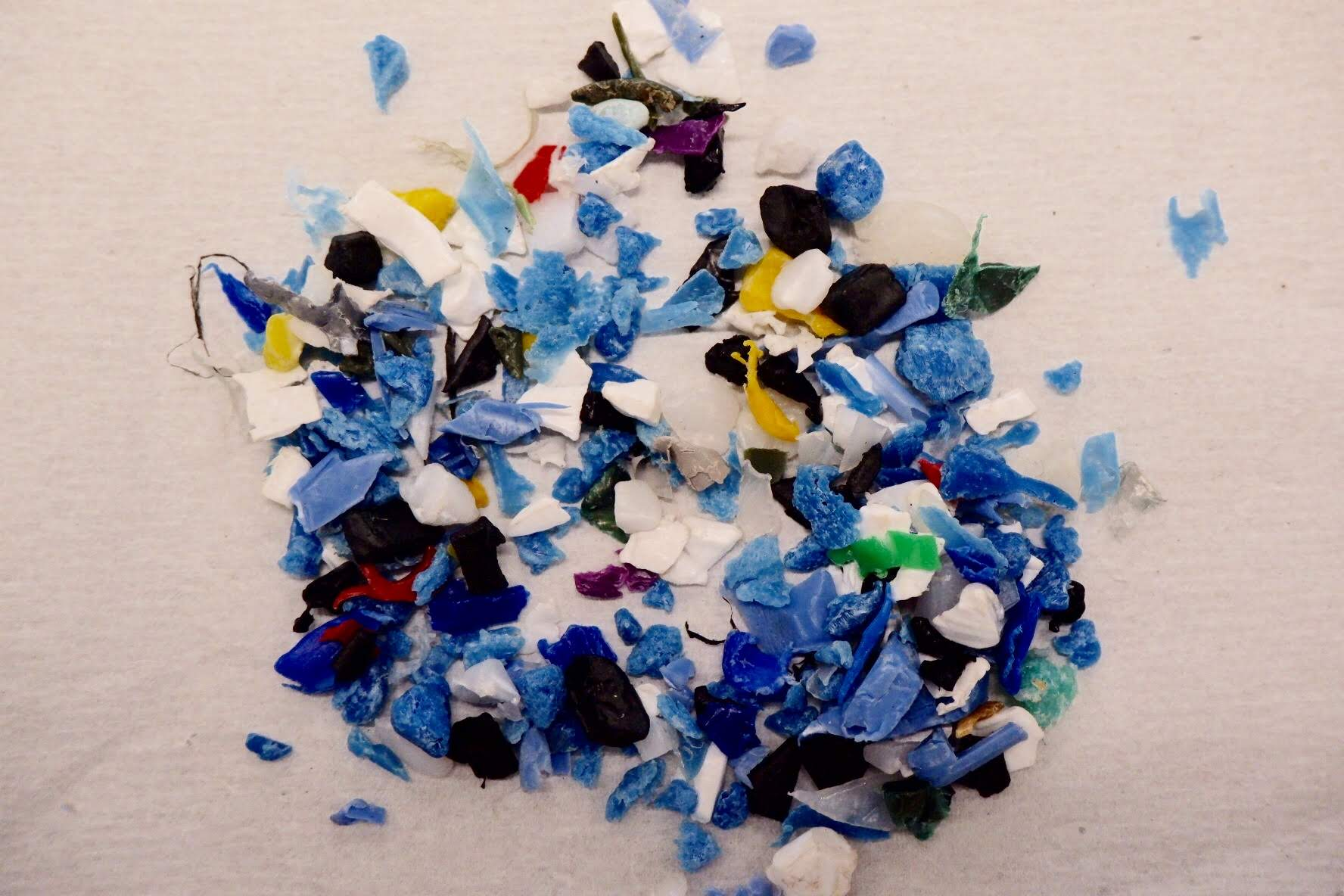


Figure S2: LDPE fragments used in this study


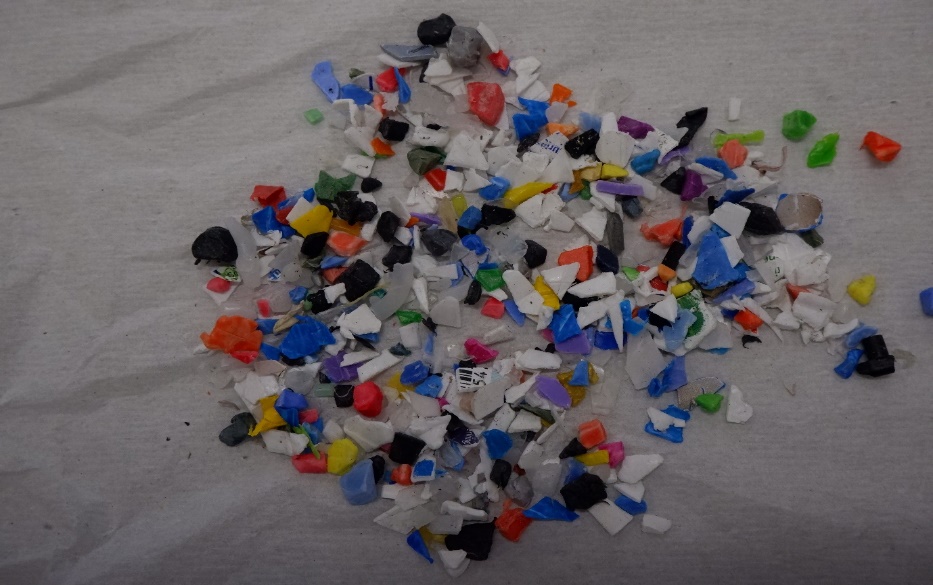


Figure S3: PP fragments used in this study

Table S2: Mass and weight of microplastic fractions used in the soil microplastic establishment experiments.

| Plastic material | Density (g/cm^3^) | Component of particle sizes (g)  [w/w] | | | | Total (g) |
| --- | --- | --- | --- | --- | --- | --- |
|  |  | **2 mm** | **1.4 mm** | **0.25 mm** | **0.045 mm** |  |
| HDPE | 0.97 | 4.0  [52%] | 1.5  [20%] | 2.00  [26%] | 0.15  [2%] | 7.65 |
| LDPE | 0.94 | 1.0  [35%] | 1.88  [65%] | - | - | 2.88 |
| PP | 0.91 | 5.0  [39%] | 3.0  [23%] | 4.5  [35%] | 0.09  [0.7%] | 12.59 |

Table S3: Contents of microplastics, soil and organic matter used in the soil-microplastic establishment procedure

1. **LDPE, pellets, 2.5%**

| LDPE | Soil (g) | Organic matter (g) | MP(g) |
| --- | --- | --- | --- |
| A | 20.00 | 0.8 | 0.5 |
| B | 20.00 | 0.8 | 0.5 |
| C | 20.00 | 0.8 | 0.5 |

1. **LDPE, fragments, 2.5%**

| HDPE | Soil (g) | Organic matter (g) | MP(g) |
| --- | --- | --- | --- |
| A | 20.00 | 0.8 | 0.5 |
| B | 20.00 | 0.8 | 0.5 |
| C | 20.00 | 0.8 | 0.5 |

1. **PP, fragments, 2.5%**

| PP | Soil (g) | Organic matter (g) | | MP(g) |
| --- | --- | --- | --- | --- |
| A | 20.00 | | 0.8 | 0.5 |
| B | 20.00 | | 0.8 | 0.5 |
| C | 20.00 | | 0.8 | 0.5 |

1. **Blank**

| Blank | Soil (g) | Organic matter (g) | MP(g) |
| --- | --- | --- | --- |
| A | 20.00 | 0.8 | – |
| B | 20.00 | 0.8 | – |
| C | 20.00 | 0.8 | – |

Table S3: **Soil-microplastic establishment**

Table S3A:Organic matter enzymatic digestion using PP microplastics

| Samples | Wt of glass bottle | Wt of soil (g) | Wt of Mp (g) | Wt of OM (g) | Reconditioned soil +OM+MP | Wt of contents +H2O2 | Wt of contents after H2O2 | Wt of contents + SDS | Wt of contents +after SDS | Wt of contents + C;H | Wt of contents after C;H | Wt of contents + lipase | Wt of contents after lipase | Wt of contents+ protease | Wt of contents after protease | Wt of contents + H2O2 | Wt of contents after H2O2 | Final Wt of OM+ MP (dry weight) | Digestion  efficiency |
| --- | --- | --- | --- | --- | --- | --- | --- | --- | --- | --- | --- | --- | --- | --- | --- | --- | --- | --- | --- |
| PP_A_ | 257.11 | 20 | 0.5 | 0.8 | 288.25 | 320.38 | 314.76 | 311.62 | 311.45 | 325.24 | 325.1 | 324.63 | 324.38 | 328.17 | 327.17 | 320.43 | 318.73 | 0.71 | 83.8% |
| PP_B_ | 251.84 | 20 | 0.5 | 0.8 | 282.89 | 315.15 | 309.58 | 308.43 | 308.38 | 317.83 | 317.6 | 335.43 | 334.79 | 326.28 | 326.05 | 316.38 | 314.45 | 0.59 | 93.1% |
| PP_C_ | 257.08 | 20 | 0.5 | 0.8 | 288.15 | 320.23 | 314.84 | 307.7 | 307.42 | 323.84 | 323.56 | 324.99 | 324.8 | 330.83 | 330.53 | 323.25 | 321.36 | 0.73 | 82.3% |

| Samples | Wt of glass bottle | Wt of soil (g) | Wt of Mp (g) | Wt of OM (g) | Reconditioned soil +OM+MP | Wt of contents +H2O2 | Wt of contents after H2O2 | Wt of contents + SDS | Wt of contents +after SDS | Wt of contents + C;H | Wt of contents after C;H | Wt of contents + lipase | Wt of contents after lipase | Wt of contents+ protease | Wt of contents after protease | Wt of contents + H2O2 | Wt of contents after H2O2 | Final Wt of OM+ MP (dry weight) | Digestion  efficiency |
| --- | --- | --- | --- | --- | --- | --- | --- | --- | --- | --- | --- | --- | --- | --- | --- | --- | --- | --- | --- |
| LDPE_fA_ | 260.86 | 20 | 0.5 | 0.8 | 291.88 | 324.15 | 318.6 | 309.64 | 309.39 | 328.65 | 328.26 | 327.69 | 327.5 | 334.3 | 334.16 | 325.75 | 324.22 | 0.84 | 73.8% |
| LDPE_fB_ | 253.29 | 20 | 0.5 | 0.8 | 284.35 | 316.75 | 311.13 | 300.32 | 300.16 | 321.2 | 320.7 | 317.15 | 317.04 | 327.66 | 327.49 | 318.72 | 316.88 | 0.81 | 76.2% |
| LDPE_fC_ | 247.66 | 20 | 0.5 | 0.8 | 278.43 | 309.94 | 304.49 | 300.01 | 299.92 | 316.15 | 316.08 | 313 | 312.79 | 319.3 | 319.18 | 304.34 | 302.57 | 0.92 | 67.7% |

Table S3B: Organic matter enzymatic digestion using LDPE fragments

Table S3C: Organic matter enzymatic digestion using LDPE pellets

| Samples | Wt of glass bottle | Wt of soil (g) | Wt of Mp (g) | Wt of OM (g) | Reconditioned soil +OM+MP | Wt of contents +H2O2 | Wt of contents after H2O2 | Wt of contents + SDS | Wt of contents +after SDS | Wt of contents + C;H | Wt of contents after C;H | Wt of contents + lipase | Wt of contents after lipase | Wt of contents+ protease | Wt of contents after protease | Wt of contents + H2O2 | Wt of contents after H2O2 | Final Wt of OM+ MP (dry weight) | Digestion  efficiency |
| --- | --- | --- | --- | --- | --- | --- | --- | --- | --- | --- | --- | --- | --- | --- | --- | --- | --- | --- | --- |
| LDPE_A_ | 254.8 | 20 | 0.5 | 0.8 | 285.73 | 315.82 | 310.52 | 307.11 | 307.06 | 320.54 | 320.28 | 322.34 | 322.09 | 326.85 | 326.58 | 314.95 | 313.79 | 0.63 | 90.0% |
| LDPE_B_ | 256.55 | 20 | 0.5 | 0.8 | 287.77 | 319.82 | 314.15 | 305.45 | 305.35 | 321.98 | 321.8 | 325.07 | 324.89 | 324.04 | 323.81 | 318.7 | 316.65 | 0.52 | 98.5% |
| LDPE_C_ | 260.94 | 20 | 0.5 | 0.8 | 292 | 321.75 | 316.68 | 313.45 | 313.39 | 325.82 | 325.62 | 329.04 | 328.94 | 333.29 | 333.14 | 322.91 | 321.51 | 0.62 | 90.8% |

Table S3D: Organic matter enzymatic digestion using Blank samples

| Samples | Wt of glass bottle | Wt of soil (g) | Wt of Mp (g) | Wt of OM (g) | Reconditioned soil  + OM+ MP | Wt of contents +H2O2 | Wt of contents after H2O2 | Wt of contents + SDS | Wt of contents +after SDS | Wt of contents + C;H | Wt of contents after C;H | Wt of contents + lipase | Wt of contents after lipase | Wt of contents+ protease | Wt of contents after protease | Wt of contents + H2O2 | Wt of contents after H2O2 | Final Wt of OM+ MP (dry weight) | Digestion  efficiency |
| --- | --- | --- | --- | --- | --- | --- | --- | --- | --- | --- | --- | --- | --- | --- | --- | --- | --- | --- | --- |
| BLANK_A_ | 247.1 | 20 | 0.5 | 0.8 | 277.7 | 309.77 | 304.16 | 294.35 | 294.27 | 312.23 | 312.04 | 312.78 | 312.64 | 316.03 | 315.8 | 306.92 | 305.33 | 0.05 | 93.8% |
| BLANK_B_ | 256.14 | 20 | 0.5 | 0.8 | 286.73 | 318 | 312.48 | 306.05 | 305.94 | 319.06 | 318.87 | 322.41 | 322.24 | 328.49 | 328.34 | 316.92 | 315.01 | 0.04 | 95.0% |
| BLANK_C_ | 257.76 | 20 | 0.5 | 0.8 | 288.35 | 320.55 | 314.86 | 310.82 | 310.69 | 320.26 | 319.97 | 322.78 | 322.58 | 327.14 | 326.93 | 319.05 | 317.64 | 0.04 | 95.0% |
